# Supplementary material for: “Sometimes I think my frustration is the real issue”: A qualitative study of parents’ experiences of transformation after a parenting programme
Source: PLoS One. 2021 Oct 12;16(10):e0258528. doi: 10.1371/journal.pone.0258528 (PMC8509979; doi:10.1371/journal.pone.0258528)
Supplement: Attachment — Submitted filename: McKayKennedyYoung-RevisionResponse.docx [file pone.0258528.s001.docx]

Dear Dr Warner-Czyz,

Thank you so much for the opportunity to revise our paper. We are grateful for the thoughtful reviews that were given.

Please find our revisions and responses below, as well as the attached revised paper. We hope our revisions have made the paper stronger and suitable for publication.

**If there are ethical or legal restrictions on sharing a de-identified data set, please explain them in detail (e.g., data contain potentially identifying or sensitive patient information) and who has imposed them (e.g., an ethics committee). Please also provide contact information for a data access committee, ethics committee, or other institutional body to which data requests may be sent.**

Although we removed place and person names from transcripts, these are verbatim records of in-depth interviews and contain details of the family living arrangements and structure which could the identification of some families. We would therefore invite researchers with interests in accessing the data to contact Dr Jane Petty, who is the overall research coordinator for the Tavistock’s Research & Development team. Her email is [j.petty@tavi-port.nhs.uk](mailto:j.petty@tavi-port.nhs.uk).

**Reviewer #1: Summary of the Research**

**The manuscript used a qualitative approach to analyze themes in parent interviews regarding parent perceptions of participating in an Incredible Years (IY) parent training program. The authors claim that parents improved their understanding of their children and themselves after participating in parent training. Additionally, the authors claim that participants in the first two groups reported feeling calmer and more confident as parents.**

**Strengths Overview**

**The manuscript highlights the perceptions of parents who did or did not attend a parenting program. The introduction highlights the importance of understanding parents' motivation for change and positions the study as a mechanism for understanding parents' motivation for change during and after participation in a parent training program. Using a qualitative approach to inquiry allows for a rich and in-depth exploration of the phenomena in context. The manuscript contains several relevant quotations to support the arguments of the authors. The topic of the current study is relevant to the current literature and could help expand the current knowledge about the perceptions toward parent training programs of parents who dropped out, did not attend, or whose child behaviour scores did not improve after completing the IY parent training curriculum.**

Thank you for these observations.

**Weaknesses Overview**

**The manuscript has several limitations that should be addressed. The manuscript lacks a clear research objective as the description is vague. The authors should clarify the following sections to avoid confusion. Additionally, the authors make causal claims that are not appropriate given the qualitative nature of the study.**

**Abstract**

**1. The problem under investigation is not described in the abstract.**

This has been rectified in the ‘Introduction’ section of the revised abstract, page 2.

**2. Clarification as to the type of parent programme (i.e., behaviour management training) would be beneficial in the abstract.**

The clarification “Parenting programmes help to alleviate conduct problems in children, but ensuring that all parents feel supported to attend, complete and learn from these programmes has proven difficult.” has been added to page 2, lines 28-30.

**3. The authors should clarify whether the causal attribution made in the second to last sentence is referring to the parent's perception of the problem or study findings. The wording in that sentence suggests that this causal relationship was demonstrated in the study.**

The ‘Discussion’ section of the abstract has been revised, pages 2-3.

**Introduction**

**4. The introduction nicely explains that positive parent-child interactions are beneficial for children and their parents. However, the link between positive parent-child interactions, positive parenting (mentioned on p. 3, first sentence of second paragraph), and parenting programs is not clear in the text. Consider explicitly stating the link between these variables in order to bring context to your study.**

The context and connection between the first two paragraphs has been strengthened by inserting “Positive parenting practices are associated with better outcomes for children, such as decreased externalizing problem behaviour (Weeland et al., 2017)” on page 4, lines 95-6.

**5. The second sentence of the first paragraph on page 3 connects two thoughts with the word "whereas." However, the wording of the two thoughts does not make them seem contradictory. Clarification is needed to understand the idea that the authors are attempting to convey in this sentence.**

We hope this revision provides clarity: “Improved parent-child interactions are associated with parents feeling more competent when managing their child’s behaviour (de Haan et al., 2009), whereas parents who believe they have little control over their child’s ‘difficult’ behaviour report feelings of hopelessness and incompetence (Sevigny and Loutzenhiser, 2009)” on page 4, lines 112-116.

**6. A brief description of parent training programmes in general would better frame the context of the study.**

We now describe these as follows: “Parenting programmes, often facilitator-led and focused on enhancing parents’ knowledge and skills around their child’s development and behaviour, are effective interventions for children demonstrating difficult behaviours (Leijten et al., 2018; Waddell et al., 2018)” has been added on page 4, lines 104-106. Relevant references have also been added to the paper.

**7. In addition to your review, consider providing a critique or synthesis of the applicable literature to identify key issues in the relevant literature and specify knowledge gaps in current literature. In other words, make it very clear that your study will fill in a gap in the literature after describing the current gap in knowledge.**

We hope this additions clarifies: “However, there is little other evidence to draw on regarding how parents who have attended parenting programme are enabled to make changes in both a perceptual and practical sense” on page 6, lines 136-137. Revisions made to the abstract and discussion also help to add clarification.

**8. There are no clearly stated research questions and the study objective is vague. After reading the introduction, I was still unsure as to what exactly the study was directly aiming to accomplish. Without specific research questions or objectives, one cannot evaluate if the approach was appropriate for addressing the research objectives.**

We hope this reworking adds clarity: “We sought to better understand the process of change as experienced by parents following a parenting programme. This article examines their narratives of change, that is, how and why parents felt motivated to change, and how they made these changes occur in their everyday lives. Developing a parent-centred understanding of change could identify ways that programmes might enhance their support for parents, with the ultimate goal of enabling more families who do not currently benefit from parenting programmes to do so” on page 6, lines 143-149.

**9. Qualitative research allows for insight into the perception of participants. Consider providing a brief rationale for using a qualitative approach to address the research objectives.**

A rationale has been provided in two places.

Page 6, lines 138-141: “Qualitative work examining the process of change from the perspectives of patients is increasingly helping in adult psychotherapy research by providing insights to enhance how therapists understand and respond to patients (Levitt et al., 2016), but work of this sort in the context of parenting programmes is limited”.

(Levitt, H.M., Pomerville, A. and Surace, F.I., 2016. A qualitative meta-analysis examining clients’ experiences of psychotherapy: A new agenda. Psychological bulletin, 142(8)).

Page 7, lines 161-163: “The qualitative study broadly sought to “improve understanding of how programmes led to good outcomes for some parents, and poor outcomes for others” from the perspective of parents (McKay et al., 2020)”.

The references have also been added to the reference list.

**Methods**

**10. Provide a rationale for fit of design used to investigate the study purpose.**

This has been added on pages 6 and 7, as described above.

**11. In the first paragraph of Page 7, the manuscript describes the different parent groups and states that the groups were based on "children who showed improved behaviour" and "children who showed worsening behaviour." It is unclear how the child's behaviour was measured. The wording suggests that the change in behaviour was demonstrated by an observation ("showed") but it should be made clear. Although this change in behaviour is not the main variable of interest in the manuscript, a brief description would be helpful to understand the context of the study.**

A sentence has been reworked on page 9, lines 200-204, which hopefully provides clarity: “Scores on the Parent Account of Child Symptoms (PACS; Taylor et al., 1986), a structured parental interview on children’s behavioural issues (that was part of the quantitative phase and completed before and after the recent IY), formed the basis of assessing whether a child’s behaviour had improved, worsened or was unchanged since the parenting programme”.

**12. Given that generalizability to large populations is limited in a qualitative study, it would be helpful for the reader to understand a little more about the participants' demographics and cultural information.**

While the study does not aim for generalisability, this paragraph has been adjusted to ensure contextualisation of the sample (page 9, lines 209-215): “From information gathered at the time of the interview, of the 42 participants, there were 36 mothers, two fathers, two grandmothers, and two other primary caregivers, caring for 27 male children and 15 female children. The children’s average age was 6.7 years old (range 4-10 years). Twenty-four of the parents had a partner, and 18 were single, but the narratives indicated recent fluctuations in the relationship status of some participants over the course of the PPC project. Care was also taken to ensure a socio-economically and ethnically diverse sample”.

**13. When describing the sample, it would be helpful to be specific about the time at which this information was gathered. For example, in the first paragraph of Page 7, the manuscript describes the relational status of the parents. However, it refers to fluctuating relationship statuses over the course of the project, so it is not clear at which point the authors are describing the sample. At the time of the start of the project, during the project, or at the time of the interviews?**

“From information gathered at the time of the interview” has been added at the beginning of the paragraph (page 9, line 209) to avoid any confusion.

**14. It would be beneficial for the reader to understand the specific purposive selection process method used and inclusion/exclusion criteria.**

The specific purposive selection process in detailed throughout the ‘Recruitment procedures and participant sample’ sub-section over pages 7-10.

**15. Briefly describe the coders or analysts and their training.**

We have now elaborated this on page 10, line 246-248, to indicate that KM, who also did the bulk of the interviews “has an extensive background in qualitative research in mental health and has received and delivered training in this area, a second interviewer SP has a background in health-related research and has received training in qualitative methods”.

**Results**

**16. The authors should clarify whether the causal attributions made in the results section are referring to the parent's perception of the problem, empirical research findings, or author's interpretation of study findings. The wording of some sentences suggests that causal relationships were demonstrated in the study. Consider adding a citation to causal statements, including evidence for the statement, or rewording the sentences to make them more accurate. A list of some causal statements follows:**

**○ Page 13, first paragraph: "By helping parents to ground their parenting in empathy, IY and other parenting programmes changed the way these parents interacted with their children."**

**○ Page 17, second paragraph: "Kindness could change every-day interactions with children too."**

**○ Page 20, first paragraph: "Parenting programmes helped to remind other participants that their children could not necessarily understand things or act in the same way as adults."**

We have tried to make it clearer in the results section that we are presenting parents’ perspectives on how they changed and what helped them to change. For example, near the start of the results section we refer to how what parents “spoke about” and what “parents felt differently about”. We have also reworded the specific examples the reviewer raised to clarify that these were referring to the parents’ perceptions.

Page 15, lines 369-371: “By helping them to ground their parenting in empathy, Group 1 and 2.1 parents felt that learnings from IY and other parenting programmes had enabled them to change the way they interacted with their children”.

Page 20, lines 484-486: “Parents also felt that kindness could change every-day interactions with children too. Since attending the programmes some parents described realising that they could often sound grumpy towards her children, even if they were not feeling that way”.

Page 20, lines 552-553: “Parenting programmes appeared to help parents keep in mind that their children could not necessarily understand things or act in the same way as adults”.

**Discussion**

**17. The authors should describe how the current study findings are similar and different from prior research findings.**

**18. Consider describing the types of contributions made by the current study findings in relation to previous research (e.g., challenging, elaborating on, or supporting prior research).**

In order to respond to both 17 and 18, we have significantly revised the Discussion, pages 29-32.

**19. Consider describing how the study findings can be utilized by practitioners and or researchers.**

Additions have been made in the discussion to more clearly describe how study findings can be used.

Pages 32-33, lines 793-795: “Facilitators of future programmes should look to see how their practice could better support reflection by parents and potentially help more families to benefit from programmes”.

Page 31, lines 748-761: “Positive reinforcement, whether from seeing their child’s behaviour improve, feeling calmer or from facilitators, helped parents to engage with the parenting programme even if they had been hesitant to start with. The reported absence of encouragement from the facilitators as noted by parents in Groups 2.2 and 3 highlights the importance of attending to the differences between how programmes are intended to be delivered and how they are experienced by parents. This study did not involve direct observations of programmes being delivered, and so we do not know how far the narratives of parents, including those in Groups 2.2 and 3, reflect what actually happened in sessions. However, the reported lack of facilitator encouragement from parents in Groups 2.2 and 3 suggests that at best, encouragement was not provided in ways that were meaningful for these parents. More broadly, our findings also demonstrate the importance of parents having agency within a parenting programme (Levitt et al., 2016), where they feel enabled to explore, with guidance and encouragement and from facilitators, what strategies and approaches fit within their own family dynamic”.

**Limitations and Conclusion**

**20. The authors claim to demonstrate that there is a causal relationship between a parent's increased understanding of themselves and their child and an increase in parent-child interactions (see p. 28, first sentence of second paragraph in the Discussion section). The authors state: "The findings indicate that when parenting programmes helped parents to better understand themselves and their child, this led to more positive parent-child interactions, and a more empathetic relationship." However, the data and methods do not fully support this conclusion. Specifically, a qualitative study cannot prove the causal influence of one variable on another. Additionally, the variables in this sentence are not defined. For example, "a parent's increased understanding of themselves" indicates that there is an increase from a baseline measurement of parental understanding of themselves. The question of how the authors know that understanding and empathy increased from before the program started is left unanswered. Understanding and empathy are difficult constructs to measure. Parent-report of these constructs should be clearly identifiable as such.**

We agree that the qualitative data we present do not enable conclusions to be drawn about cause-effect relationships in the way that evidence from RCTs would. Nor is the study comparing variables. Rather akin to qualitative work on adult psychotherapy reviewed by Levitt et al, 2016 (which we have now referenced), our article is reporting on parents’ perceptions and lived experience of how parenting programmes enabled them to change. Here the importance is placed on how a parent talks about their experience of change, how they felt their child’s behaviour has improved, as opposed to their responses being measured externally. In line with Levitt’s findings, sensitivity to the parents’ experiences is prioritised.

We have reviewed and revised our wording throughout the discussion, using phrasing from the outset that makes it clear that we are reporting on parents’ perceptions of what helped them and how change came about in their eyes. For example, “The findings indicate that when parents felt that a parenting programmes helped them to better understand themselves and their child, this led to more positive parent-child interactions, and a more empathetic relationship” on page 32, lines 786-788.

**21. The last sentence of the manuscript is confusing which limits the readability of your conclusion.**

This has been reworked: “From the perspective of parents, programmes that enabled them to reflect on the ways they responded to their child helped them to incorporate what they had learnt into their everyday lives, and improved the lives of families regardless of whether the child’s behaviour had improved. Facilitators of future programmes should look to see how their practice could better support reflection by parents and potentially help more families to benefit from programmes” on pages 32-33, lines 790-796.

**References**

**22. The authors should review the References section of the PLOS ONE Submission Guidelines and the NIH website with International Committee of Medical Journal Editors (ICMJE) Samples of Formatted References (https://www.nlm.nih.gov/bsd/uniform_requirements.html) for information regarding the references. Specifically, the authors should:**

**○ Place a colon after the volume number**

**○ Format the page numbers according to the guidelines (e.g., remove "pp." on page 30)**

**○ Remove the extra year from the fifth reference on page 30**

All relevant changes have been made.

**Grammatical and Stylistic Review**

**1. The first letter in section heading words should be capitalized (i.e., "Limitations and conclusion" header on page 28).**

These have all been capitalised.

**2. Some of the sentences are wordy, making it difficult to follow. I advise the authors work to improve the flow and readability of the text (e.g., run-on sentences should be reworded or separated into two sentences, replace wordy sentences with concise statements, etc.). For example, the last sentence of the second paragraph on page 4 can be condensed by removing the first six words of the sentence. There is no need to mention the reference twice in the same sentence. The additional citation takes away from the readability of the text.**

Changes as suggested have been made throughout the revised manuscript.

**3. Consider replacing "our study" with "the current study" throughout the document.**

**4. Consider replacing “we” with “the researchers” throughout the document.**

We have limited the use of ‘our study’ and replaced this in several places with ‘the current study’ or ‘this study’. However, many prominent biomedical journals advise using the first person and active voice for clarity (please see <http://www.biomedicaleditor.com/active-voice.html>) and so we have retained ‘our study’ and ‘we’ in some instances to maintain clarity.

**Reviewer #2: This study explores the perspectives of participants / nonparticipants in an Incredible Years (IY) program. The data were collected in conjunction with a larger multi-phase, mixed methods study which is ongoing. As part of this qualitative study, 42 participants were interviewed about their experiences and the data subjected to analysis through a phenomenological lens. This is an impressive sample size in a qualitative study. Three groups of participants were distinguished: (1) participants in IY programs who reported improved behaviour in their child, as indicated on a quantitative measure (Parent Account of Child Symptoms; PACS); (2) participants in IY programs whose children showed no change or worsening behaviour as measured by the PACS; (3) nonparticipants or participants who had dropped out of the IY program.**

**As the authors note, the perspectives of parent/ caregiver participants are an important source of information about parent groups designed to address and, hopefully, improve behavioural concerns in their children. In the paper, a number of compelling and informative quotes from participants in the study are included as part of the data reported, and attest to how powerful information gleaned from qualitative research can be. The size of the sample in the study and the detail in some aspects of the methodology reported are strengths of the paper. For example, details about the interviewers and the interviews including the use of recordings, transcription and field notes are noteworthy and consistent with SRQR and COREQ standards utilized by PLOS in evaluating qualitative research.**

Thank you very much for these comments.

**I would recommend that the specific questions used to guide the interviews also be included in the paper for further information.**

A summary of the interview questions is listed on page 10, lines 238-245: “Interviewers began by asking the parent to describe their child and, to maintain a positive frame, usually ended by asking parents to describe ‘a good day’. Questions were open-ended and structured around family life prior to IY, how and why parents had been referred to IY, or about their perceptions of parenting programmes more broadly for those parents who had not been referred. We also explored parents’ experiences of attending IY (where applicable), including any perceived benefits and what they would change about the programme, other support they had accessed, and whether family life had changed since IY, or over the equivalent timescale for those in Group 3”.

**In addition, it might be helpful to include the background and training of the interviewers as well, given the clinical nature of the programs which are being explored.**

This has been clarified on page 10, lines 246-248: “KM has an extensive background in qualitative research in mental health and has received and delivered training in this area, a second interviewer SP has a background in health-related research and has received training in qualitative methods”.

**In addition, a general description of the settings in which the programs are delivered (e.g., community, school, hospital) might be helpful.**

This has been clarified as “community-based” on page 8, line 182.

**However, I have the following general comments about information presented in the paper.**

**In the Introduction, the rationale for conducting the study needs to be stated more explicitly. It is not clear from the Introduction why the study was conducted and what the qualitative research methodology contributes to that research question. Interesting research in the area is reviewed but it needs to be integrated, and how this research relates specifically to the study needs to be more explicitly stated. There are references to the findings and perspectives of several researchers and it would be helpful to describe the specific nature of their contributions and how these contributions informed the rationale and interpretation of the findings of the current study.**

This has been clarified throughout the revised paper (see also responses to reviewer one), particularly on page 6 of the Introduction and page 7 of the Methodology.

**In addition, in the Introduction, there should be a description of IY programs, including goals, content and process of program delivery.**

This has been clarified on pages 7-8, lines 172-175: “Developed by Webster-Stratton, and delivered by trained facilitators, IY “is a comprehensive set of programs designed to promote social competence and prevent, reduce, and treat aggression and related conduct problems in young children (ages 3 to 10 years)” (Webster-Stratton, 2001, 32)”.

**In the design, as presented, all participants were recruited as part of participation in the IY programs, although participants in Group 3 either did not complete, or attend, the programs. The IY program is an important reference in the design and needs to be included in the interpretation of results - understanding that parents may often access multiple resources (as noted for Group 3): participants were recruited from them, and 34 participants did attend IY programs. There was also little discussion about why many caregivers in Group 2 reported improvements in their children’s behaviour, which was not seen on the quantitative measure. There are a number of potential explanations for this, which should be explored. For example, this difference could have implications from how change is measured or how outcomes are defined in future research, and speak to information which qualitative research can provide in understanding change.**

As stated on page 7, lines 170-172, recruitment of participants was not only based on participation in the IY programme, but “if they had attended, been referred, or been considered for referral, to an Incredible Years (IY) parenting programme in three sites in south east England”. This is an important difference as, while every parent had done some form of parenting programme in the past, not every parent in our study had participated in or completed IY.

Further, the findings in this article focused on the how parents perceived an improved family environment, in themselves and/or in their child, even if this positive change was not reflected in the quantitative scores. Unpacking the reasons for the differences between the qualitative data on change and quantitative scores is outside the remit of this particular article, however we identify it as important for future studies: “From the perspective of parents, programmes that enabled them to reflect on the ways they responded to their child helped them to incorporate what they had learnt into their everyday lives, and improved the lives of families regardless of whether the child’s behaviour had improved. Facilitators of future programmes should look to see how their practice could better support reflection by parents and potentially help more families to benefit from programmes” (pages 32-33, lines 790-795). We hope as well that the revised conclusion (pages 32-33) helps to address some of the uncertainty.

**In the results section, 3 themes are presented: Parents better understanding themselves; Parents better understanding their children and Calmer parent, calmer child. The experience of parents is described as ‘transformative’ (page 10), and it would be helpful if this construct were defined before themes are presented. Within each of these themes, presentation of the subthemes, and how they relate to the main theme needs to be made more explicit so that the theme is clear to the reader. In addition, the relationship of each quote to the theme needs more clarity.**

‘Transformative’ has been clarified on page 13, lines 301-303 as “where they felt differently about themselves and their child as a result of the parenting programme compared to before they started”. We have also delineated sub-themes within two of the themes explored:

1.	Better understanding themselves

a.	Reflecting on actions and decision-making

b.	Who has to change?

c.	Learning from the past

2.	Better understanding their child

a.	What could be controlled?

b.	Changes made to support the child

We have also added pseudonyms to the participant quotes as we felt this helped to contextualise the quotes and make the links between the quotes and the themes clearer.

**In the discussion, the main findings of the study need to be stated clearly, and their implications discussed. Some interesting work is presented, but is not described in sufficient detail, and its relationship to the findings in the current study is not made clear. In addition, the relationship of the findings of the study to the goals of the IY parenting programs is not discussed. While I note references to several parenting strategies in the quotes provided by participants, there is no discussion of strategies / program content or their potential implication for parents’ experiences as group participants.**

As in our response to reviewer 1 above, we have substantially revised the discussion to make the findings clearer and to better delineate the implications of the findings for parenting programmes.

**Minor comments for consideration are:**

**The format of the abstract needs to be revised**

The abstract has been revised, pages 2-3.

**In the Method section, information about the Personalized Programmes for Children (PPC) Project includes a description about design, but is limited in any information about content: is this study the qualitative phase of the early phase of the larger project?**

This has been clarified in the first section of the Methodology on page 7.

**More information about scores on the PACS which formed criteria for inclusion in Group 1 vs Group 2 should be stated**

A description is given on page 9, lines 200-204: “Scores on the Parent Account of Child Symptoms (PACS; Taylor et al., 1986), a structured parental interview on children’s behavioural issues (that was part of the quantitative phase and completed before and after the recent IY), formed the basis of assessing whether a child’s behaviour had improved, worsened or was unchanged since the parenting programme”.

**If reasons to decline participation were given, it would be helpful to include these reasons**

Detail has been added in page 9, lines 206-208: “the 10 parents who were not interviewed either could not be reached after several attempts to contact them (N=9), or declined the interview invitation (N=1)”.

**Note that 42 participants were reported, although the subcategories add up to 41**

This has been rectified on page 9, line 210. All the other sub-categories are correct.

**I appreciate the inclusion of participants who were not biological parents. However, it would be helpful to include some (non-identifying) information about the role these individuals played in children’s lives e.g., primary caregiver in the home; primarily caregiver on a daily basis etc.**

This information has been included on page 9, line 210: “there were 36 mothers, two fathers, two grandmothers, and two other primary caregivers”.

**Some information included in the Ethics section (e.g., contact with Research Assistants, followed by contact with Qualitative Interviewers) should also be included in the Method section**

The sentences “Research assistants on the quantitative phase gave parents a brief explanation and an information sheet to read about the qualitative study. If parents agreed, their contact details were shared with the qualitative team. Qualitative researchers subsequently contacted interested parents, to discuss the study further.” have been removed from the Ethics section and have been incorporated into the ‘Recruitment procedures and participant sample’ sub-section on page 8, lines 191-194

Thank you again for your helpful review of our manuscript.

Best wishes,

Kathy McKay
