# Supplementary material for: “Sometimes I think my frustration is the real issue”: A qualitative study of parents’ experiences of transformation after a parenting programme
Source: PLoS One. 2021 Oct 12;16(10):e0258528. doi: 10.1371/journal.pone.0258528 (PMC8509979; doi:10.1371/journal.pone.0258528)
Supplement: Attachment — Submitted filename: McKayKennedyYoung-RevisionResponse3.docx [file pone.0258528.s002.docx]

Dear Dr Warner-Czyz,

Thank you so much for the opportunity to further revise our paper. We are grateful for your comments and the additional reviews that were given.

Please find our revisions and responses below, as well as the attached revised paper. We hope our revisions have made the paper stronger and suitable for publication.

**Additional Editor Comments (if provided):

The reviewers and I agree the authors improved this paper from its original form based on feedback from previous reviewers. However, we feel the manuscript needs to address additional issues - both conceptual and line-level - before becoming suitable for publication**.

Thank you for these comments. We outline our responses to these points below in response to the issues outlines by both reviewers.

**Reviewer #1: Strengths Overview
The topic expands current knowledge and adds to the body of literature on parent perceptions of their parenting and children following parent training programmes. The authors did a nice job of implementing the suggestions from the prior reviews in their updated manuscript. Overall, the structure and wording has greatly improved.**

Thank you so much for your kind comments about the improvement of the paper.  **Suggestions
While the authors did a nice job of strengthening the manuscript with suggestions from the previous review (e.g., expanded upon what the IY program is about), there are some minor concerns that should still be addressed.
1. Line 120: Reword “learn what and how to change about their parenting” part of the sentence.**

This has been changed to “Completing a parenting programme can help to turn a parent’s sense of needing to change into actual changes by helping them learn how to turn intent into practice”.

**2. Line 136: Reword “how people adjust to the changes to their lives” to “life changes” in order to help the readability of the sentence.**

This has been changed.

**3. Lines 154-155: Please update the description of the IY program as the IY program is not founded upon “the premise that negative reinforcement by parents develops and maintains negative behaviours in children.” Actually, in addition to positive reinforcement, the IY program encourages parents to negatively reinforce appropriate behaviors of their children (e.g., asking politely to stop an activity, avoiding strangers, etc.). Negative reinforcement (i.e., allowing a child to avoid or escape an activity) is not the same as punishment.**

Thank you for highlighting this observation. We have now updated the description of the IY programme from the 2001 Webster-Stratton paper: “It is informed by cognitive social learning theory, and “teaches parents interactive play and reinforcement skills, nonviolent discipline techniques, including ‘‘Time Out’’ and ‘‘ignore,’’ logical and natural consequences, and problem-solving strategies” (Webster-Stratton, 2001, 35)”.

**4. Lines 267-270: consider describing the interviewers/researchers earlier in the text before referencing them. In other words, some of the information in this sentence should be presented before the interviewers are mentioned.**

This sentence has been shifted and revised: “Two researchers were present for most interviews. This was usually KM and SP: KM has an extensive background in qualitative research in mental health and has received and delivered training in this area, a second interviewer SP has a background in health-related research and has received training in qualitative methods. One researcher led the interview and the other clarified any details that may have been missed”.

**5. While the authors did a very nice job of making the writing clearer and more concise, there are still some sentences with multiple embedded clauses that interrupt the flow.
o Consider giving examples in parenthesis or breaking the sentence into two.**

The revisions made throughout the paper have assisted in making the writing more concise. We have made some specific changes throughout, including pages 4, 8, and 11.

**o Also, some of the same sentences that have multiple embedded clauses, also have missing punctuation, which makes it difficult to follow. For example, Lines 498-500 “At the time she was interviewed Olivia…”**

The punctuation has been corrected.

**6. The punctuation/wording of some sentences is not grammatically correct. For example:
o Line 338-339: “In the sections below, while we…”
o Line 381-383: “Bea, a Group 1 participant…”
o Line 567-568: “Several children in the study…” does not need a comma.
o Lines 834-836: “The findings indicate…”**

Thank you for your detailed reading. These have all been corrected.

**Reviewer #3: In relation to one of the questions above: The data (from qualitative interviews) is not made publicly available to protect confidentiality of data related to family lives and environments. The data may be accessed however on reasonable request addressed to the authors, which is fine
Other comments addressed to the authors:
This is very nice piece of work, which leads the reader to wanting to know more about the ambitious mixed-methods research project described.**

Thank you so much for these positive comments.  **Regarding the specific part reported in this manuscript, I have a few questions in relation to the methods section, and two suggestions for the discussion :
My first question refers to the choice of having a group of respondents (group 3) who had dropped out or not attended an YI programme at all? As the objective of the study was to explore the changes reported by parents in relation to attending a programme, the choice of recruiting parents who had not attended a programme does not seem completely justified. I wonder if it would be enough to focus on the narratives from the parents of groups 1 and 2.**

Thank you for raising this important point. The overall aim of the quantitative and qualitative phases of the PPC study was to develop insights on how programmes led to good outcomes for some parents and poor outcomes for others. Group 3 parents provided important insights on what might influence both initial and ongoing attendance at a parenting group. While the focus of this current paper was narrower (exploring how, from the perspective of parents, the current programmes altered their perceptions and enabled them to make positive changes in their parenting - see page 8), we would prefer to retain Group 3 in the more specific analysis. Prior to IY groups that were the focus of the PPC study Group 3 parents had previously attended other parenting groups and described changes in relation their attendance at the previous parenting groups. To clarify this, we have now amended the following sentence on page 11 to read: “It must be noted though that every parent in the sample including Group 3 had attended sessions of at least one parenting programme, whether it was in the past or the IY groups included in this study”.  **My second question is about the 200 pounds reward/incentive to participate in the study. It is not entirely clear to me if this was to participate in the qualitative interviews only or for the whole study. From my own experience of conducting research it seems a lot of money, and I would like to invite the authors to give a bit more details: how and when this was presented to the parents, why they think this was justified or necessary, how they think this might have influenced participation.**

These are important points. We agree that £200 is a significant amount of compensation for participation in the interview. However, many parents we approached to be part of the PPC study were from underserved communities in terms of access to services as well as research. We anticipated that many of the parents would feel they were not listened to or valued, and we wanted to create a safe space for them to talk. We felt offering a valuable incentive would indicate how much we respected and wanted to hear parents’ perspectives, and that we valued the time they took to speak to us, when time was a particularly stretched commodity for them. However, we took great care to ensure that the money was not coercive. The compensation amount was not mentioned by the researchers while organising the interview and was not discussed until the parent had read through the information sheet and signed the consent form. For the vast majority of the parents, the money was a complete surprise which led to some touching moments. The researchers further stressed to the parents that the compensation was a token of thanks for the time they took to participate, and that they were free to say whatever they wanted about the parenting programme and their experiences. This is demonstrated to some extent in this paper, but to a greater extent in our previous paper on the personalisation, where parents spoke about both positive and negative experiences of parenting programmes and were at times brutal in their observations of the parenting programmes. We have added more detail in the ethics section on page 13: “Regardless of how long the interview lasted, each participant received £200 worth of vouchers to demonstrate respect for parents’ perspectives and to recognise the time they gave to the study. In line with the study’s focus on voluntary participation, great care was taken to ensure the vouchers were not presented in a way that might have been perceived as coercive or that they in any way affected what parents felt they could say”.  **Unless I missed the information which might be presented as an appendix elsewhere (?), I did not see many details about the sample. In particular, some** **socio-economic or cultural characteristics would be useful, as well as some information regarding the health status of the participating parents (if relevant... it is mentioned in the results that one participant had had depression, which surely influences how child behaviours might be perceived), and whether they were professionally active when participating in the programme and later in the interviews. I understand that the sample was purposively diversified. It would be interesting to know if and how the authors looked at possible influences of some personal characteristics on the benefits associated by the parents with their participation in the programme.**

We apologise for this lack of detail, but this information was not systematically gathered for the qualitative component of the PPC study. This has been clarified on page 10: “Care was also taken to ensure a socio-economically and ethnically diverse sample, however data on socio-economic status or ethnicity were not collected in the qualitative study”.

Where parents spoke about how different life experiences impacted on their participation in the parenting programme, this has been included as context for any quotes. However, life experiences outside of the parenting programme played a more important role in a paper examining how parenting programmes could be personalised (McKay K, Kennedy E, Senior R, Scott S, Hill J, Doolan M, et al. Informing the personalisation of interventions for parents of children with conduct problems: a qualitative study. BMC Psych. 2020;20:513, https://doi.org/10.1186/s12888-020-02917-1).  **Last: one interview lasted only 16 minutes. It would be interesting to know a bit more about why this interview was so short and why the authors still considered it worthwhile to include it in their dataset.**

This is a really interesting point. It is common practice to include all interviews undertaken in the dataset during analysis. The findings from the analysis undertaken on the narratives collected have formed the basis of several papers at various stages of publication. In the case of the 16 minute interview, the parent’s experience was a straight-forward one of being referred to IY but not being able to attend any sessions of this particular group because it was impossible for them to get to the location in time and take their other children to school. This interview highlighted how important practical considerations were when referring a parent to an IY group, and formed part of one of the themes in a paper published previously from the study (McKay K, Kennedy E, Senior R, Scott S, Hill J, Doolan M, et al. Informing the personalisation of interventions for parents of children with conduct problems: a qualitative study. BMC Psych. 2020;20:513, https://doi.org/10.1186/s12888-020-02917-1). Not every interview will speak to every theme or experience, or be specifically relevant to every paper published from a study, but to not mention it as part of the analytical process felt disingenuous.

**Regarding my two suggestions for the discussion:
- I struggled a bit from the beginning about the presentation of parenting programmes aimed at changing child behaviours, and was wondering why the primary outcomes of such programmes should be child behaviours rather than parental behaviours, expectations, self-representations, etc., as well as parent-child interactions. It is clear from the results and later in the discussion that parental changes are among the main outcomes reported by the parents, and that changes in their perceptions of their child behaviours might be as important as the objective behaviours, or even more important. I think the assumption from the introduction that parenting programmes would change child behaviours could be further critically addressed in the discussion**

Thank you for raising such an interesting point. As we noted in our manuscript, parents should be involved, alongside other stakeholders, in determining what outcomes are used to assess the effectiveness parenting programmes. However, we would also note that our analysis started with a focus on the child’s behaviours because it had been concern about the child’s behaviour that tended to be the impetus for the parent’s referral to the parenting programme. This is where parents started their narrative before, at least in terms of the findings in this paper, moving onto how they then felt their own perceptions and behaviours had transformed as a result of attending the parenting programme. We felt that parents perceived an important link between their child’s behaviour and their own, especially within the theme of ‘calmer parent, calmer child’. We have sought to clarify this on page 31: “In this way, while their child’s behaviour may have been the impetus for the parent being referred to the parenting programme, these positive transformations came from a deeper understanding of how different parents and children could positively interact with each other”. We have also raised the question of how to better interrogate the nuances of ‘calmer parent, calmer child’ on page 34: “While the parents’ narratives in this paper were positive, it is important to monitor the child’s behaviour to ensure that they are also able to receive any appropriate support in the future. ‘Calmer parent, calmer child’ may have been enough to satisfy the parent in the short-term, but we do not yet know whether that leads to better long-term outcomes for the children themselves”.

**- Also, I would welcome some critique of positive parenting programmes as they are also believed to increase the risk of parental burn-out, in shaping models of an ideal parent, and consequently discrepancies between self-representations and ideal representations of a "good parent". While the authors acknowledge that the aim of their article was not to look at causal mechanisms that would support positive changes, it would yet be interesting to go a bit more into details of key-ingredients of such programmes that are believed to play a positive role. The authors could build on their suggestions that self-reflection and learning to let go are essential, but what are the components of a programme that seem key to achieve this?**

Thank you for these insightful comments. We have tried to ensure that our findings, and the discussion around them, have been led by the parents’ narratives, which were a mix of positive and negative experiences of the parenting groups, as well as experiences like the ones examined in this paper which were more nuanced. To further strengthen the exploration of these nuances, we have added a section in the Discussion on page 33 that explores these ideas: “It is all the more important to continue to dissect the factors within a parenting programme that lead to positive and negative outcomes for the parent, and consequently the child, given the implications for the future wellbeing of both. Children whose conduct problems are left untreated tend to have a lower quality of life in adulthood compared to children without such diagnoses (Sventivanyi and Balazs, 2018). The wellbeing of parents who repeatedly experience poor services for themselves, and their children, is also negatively impacted, leaving them less likely to continue seeking help (Koerting et al., 2013)”.

Thank you again for your helpful review of our manuscript.

Best wishes,

Kathy McKay
